# Supplementary material for: Effort and time costs influence motivational asymmetries in self-benefitting vs pro-environmental decisions
Source: Commun Psychol. 2025 Nov 24;3:166. doi: 10.1038/s44271-025-00347-x (PMC12643920; doi:10.1038/s44271-025-00347-x)
Supplement: Supplementary file 2 — Supplemental information [file 44271_2025_347_MOESM2_ESM.pdf]

Supplemental information

Effort and time costs influence motivational asymmetries in self-benefitting vs pro-environmental decisions

Boryana Todorova, Lei Zhang, Lukas Lengersdorff, Kimberly C. Doell, Jonas P. Nitschke, Paul A. G. Forbes, Sabine Pahl, Claus Lamm

1. Supplementary notes..2
2. Supplementary methods.. 2
2.1. Participant recruitment.. 2
2.2. Time-effort calibration procedure...2
2.3. Task design decision tree..4
2.4. Items for measuring climate change key beliefs and policy support.......................... 5
2.4.1. Climate change key beliefs.. 5
2.4.2. Policy support measure..5
2.5. Description of the environmental initiative...7
2.6. Specifications for the mixed effects models... 7
2.7. Specifications for the Bayesian analyses for null results..8
2.8. Computational modeling...8
2.8.1. Parameterization and priors... 8
2.8.2. Hierarchical Bayesian Modeling - posterior predictive checks......................... 10
2.8.3. Hierarchical Bayesian Modeling - parameter recovery.....................................11
3. Supplementary results.. 12
3.1. Distribution of climate change key beliefs and policy support..................................12
3.2. Distribution of political orientation and values scores...13
3.3. Additional plots for the proportions of costly decisions...14
3.4. Linear mixed models output.. 14
3.4.1. Models with the full sample.. 14
3.4.2. Models excluding participants with little to no variability in their behaviour......17
3.4.3. Models excluding people with doubts about being observed.......................... 18
3.5. Climate change key beliefs and discounting... 19
3.6. Ratings of effort and time levels pre- and post-testing session................................19
3.7. Preregistered analyses not included due to being suboptimal................................21
3.7.1. LMMs without trial number as covariate and with simpler error term...............21
3.7.2. Low-cost hypothesis...23
3.7.3. Linear regression between policy support and discounting difference.............24
3.8. Exploratory modeling analyses.. 24
References..26

1. Supplementary notes

Additional information on deviations from the preregistration

As preregistered, we assessed the psychometric properties of the climate change key beliefs to investigate whether we can create an overall score. However, the results of this analysis indicated that the calculation of an aggregated score was not sensible. The subscale on human causation was measured on a scale from 1 to 3, from "not happening/mainly natural causes" to "mainly human causes", and none of the participants selected the "not happening/mainly natural causes" option. This left us with a binary variable (see SI, Section 3.1. for the distribution of responses). Combining a binary item with the ordinal ones (which further differed substantially in scaling, e.g., 4-point, 6-point, and 9-point formats) can disproportionally affect the final score. Attempts to assess internal consistency (e.g., via Cronbach's alpha and polychoric correlations) were not reliable due to the heterogeneity in measurement level and highly skewed distributions on some of the items. Thus, using an average score of these items to test the "low-cost" hypothesis (which was part of our hypothesis 2) would be suboptimal. We report these results for transparency but abstain from interpreting them and drawing definitive conclusions about this hypothesis.

Of note, in our preregistration, we also stated that we would conduct an exploratory analysis to compare the effects of time and effort directly, which we abstained from conducting. As also shown during the piloting phase, finding indifference points for the lower and higher levels of effort for the time-effort calibration was not always possible, meaning the two types of cost were not perfectly calibrated to each other for all of the participants. In particular, finding indifference points for the lowest level of effort was not possible in 27 participants, and finding the indifference point for the highest level of effort was not possible in 29 participants. Therefore, we abstained from including trials from both tasks in the same model and investigating interactions between the type of cost and the other variables on the choice, as we originally intended, due to insufficient participants with successful calibration for both the low and high levels, rendering the comparison not meaningful.

2. Supplementary methods

2.1. Participant recruitment

Participants were recruited through the Vienna CogSciHub: Study Participant Platform (SPP), based on the Hamburg Registration and Organization Online Tool (hroot; Bock et al., 2014). In the recruiting text, there was no information relating to climate change/environmental protection to avoid attracting people with a particular interest in the topic. The study was described as a "decision-making experiment".

2.2. Time-effort calibration procedure

In order to ensure both effort and time demand are perceived as equally costly, such that the required physical effort and the waiting time are comparable at all levels, each participant engaged in a calibration procedure. On each trial of the calibration, they were asked whether they wanted to invest their physical effort (corresponding to the lowest and highest (i.e. 40%

and 80%) levels of effort, as previously determined in the effort calibration, and indicated on the pie chart), or wait a certain amount of time (between 3 and 27 seconds), see Fig S1. The amount of time they had to wait varied across trials so that we could calculate a point of indifference for choosing to wait versus exerting force for both the lowest and highest force levels. This allowed us to determine how much waiting time is necessary to be equally “costly” compared to exerting the respective force level. After finding a point of indifference for the lowest (40%) and highest (80%) levels, we calculated three equidistant values between the two we calibrated, resulting in 5 levels of waiting time in equal intervals to correspond to the 5 effort levels (which are in 10% of MVC intervals). This procedure was piloted with 13 different participants. This had shown, apart from a generally well-functioning procedure for the majority of participants, that the time-effort calibration does not work for everyone, as some participants always prefer to exert effort or always prefer to wait, which makes the calculation of an indifference point impossible. For those that consistently preferred the waiting times, we thus used high default values that start at 9 seconds for the lowest effort level and reach the maximum number of seconds for the highest effort level (i.e. 27 seconds), and for those who always preferred to exert effort, we took low default values starting with the lowest number of seconds tested (i.e. 3 seconds) and 15 seconds for the highest level of effort.

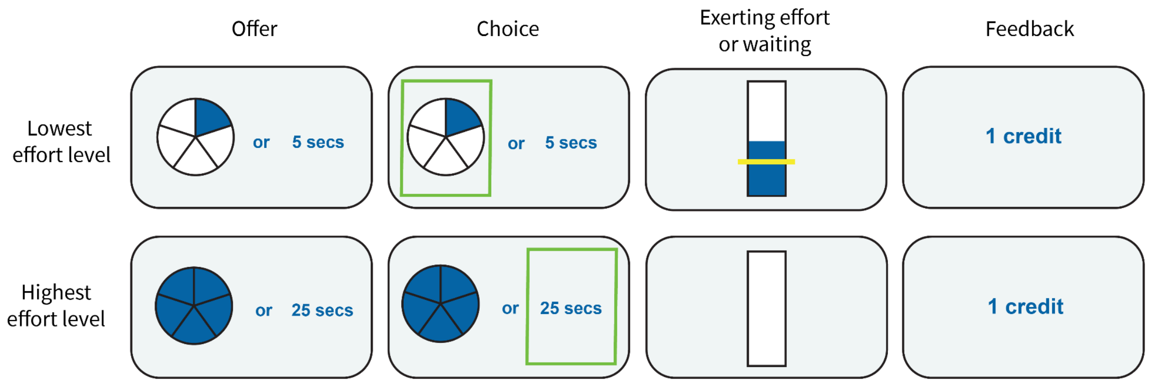

Figure S1. Calibration procedure to ensure equivalent perceived costs for effort and time. Participants chose between exerting effort (40% or 80% MVC) or waiting (3–27 seconds). Indifference points were calculated for the highest and lowest effort level (and we calculated 3 equidistant values between these two) to establish equal-cost waiting and effort times, creating five calibrated effort and time levels.

2.3. Task design decision tree

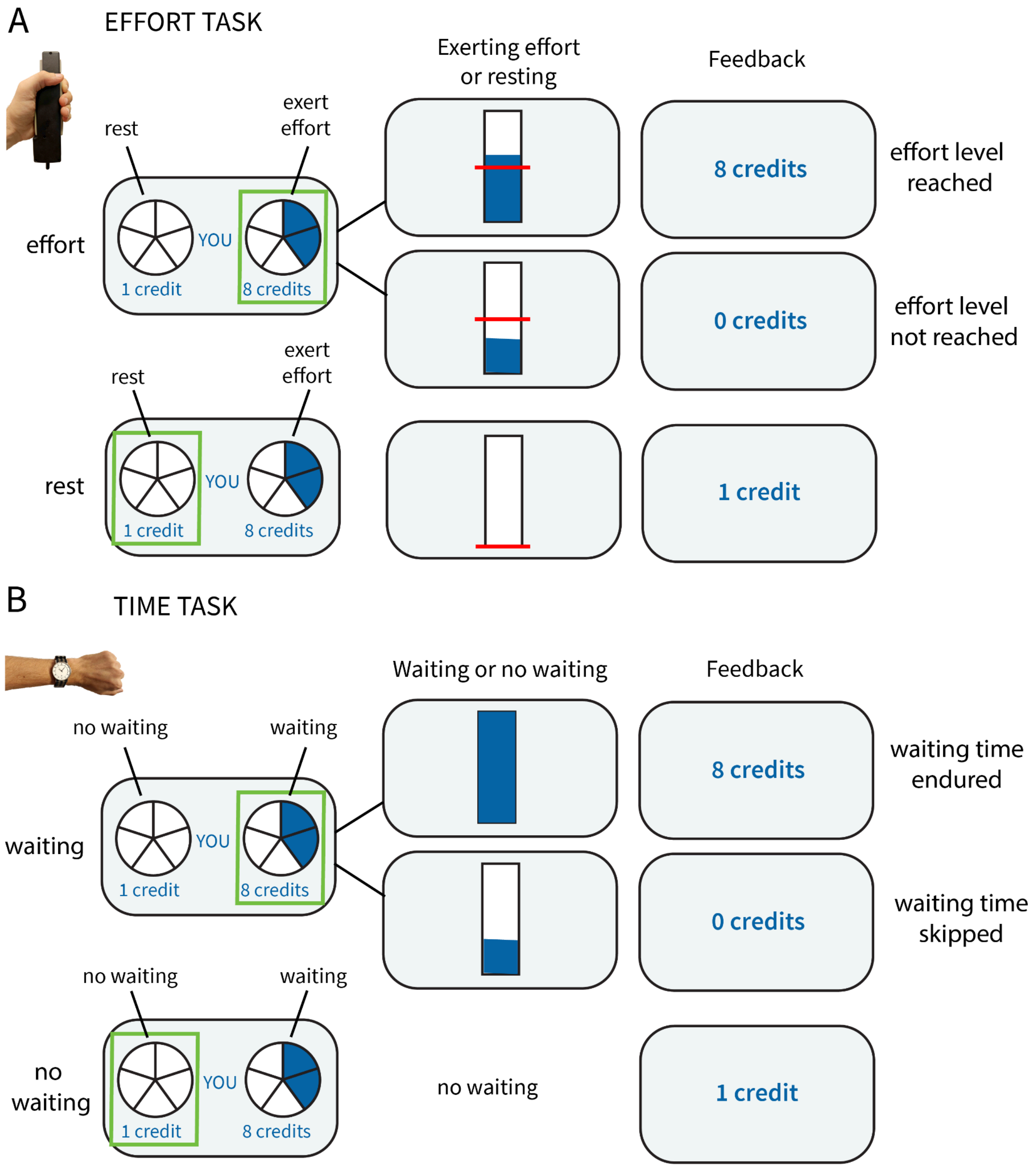

Figure 2. A. Decision tree for the effort task. On each trial, participants were given a choice between a no-effort option for a small reward and an effort option for a larger reward. If participants selected the effortful option, they were asked to exert the required force for at least 1 second within a 3-second window to earn the reward. If they did not exert sufficient effort they received 0 credits for the trial. Only self-trials selected for illustrative purposes. B. Decision tree of the time task. The task followed the same design as the effort task, with the only difference being that instead of exerting effort, participants had to endure a short waiting period. If they failed to endure the waiting period by skipping the waiting time after selecting it (analogous to failing to reach the required level of effort), they received 0 credits.

2.4. Items for measuring climate change key beliefs and policy support

2.4.1. Climate change key beliefs

Belief certainty
1-Item: "How sure are you that climate change is/is not happening?"
Measured on a 9-point scale: 1 "complete certainty that is not occurring", 5 "not certain/don't know", 9 "complete certainty that it is happening"

Human causation
1-Item, single choice: "Assuming climate change is happening, do you think it is…"

- not happening or caused mostly by natural changes in the environment
- caused by both human activities and natural change
- caused mostly by human activities

Collective efficacy
1-Item, single choice: "Which of the following statements comes closest to your view?"

- climate change is not happening or humans cannot reduce climate change even if it is happening
- humans could reduce climate change but people are not willing to change their behavior so we are not going to
- humans could reduce climate change but it is unclear at this point whether we will do what is needed
- humans can reduce climate change and we are going to do so successfully

Harm timing
2-Items:

1. "When do you think climate change will start to harm people in Austria?"
2. "When do you think climate change will start to harm other people around the world?"

Measured on a 6-point scale: never, in 100 years, in 50, in 25, in 10, right now

Harm extent
7-Items: "How much do you think climate change will harm…

- The responders themselves
- Their family
- People in their community
- People in Austria
- People in other modern industrialized countries
- People in developing countries
- Future generations

Measured on a 4-point scale: not at all, only a little, a moderate amount, a great deal

2.4.2. Policy support measure

For measuring policy support we used a novel scale developed for this study. The questionnaire had been tested on 63 participants in a prior online pilot study and reduced to 12 items (from originally 16). Standardised Cronbach's alpha was 0.87, and the item-total correlation indicated good to very good discrimination.

Participants were presented with the following text:
“To what extent would you be in favor of an EU-wide law with the following content?
If you want to behave sustainably, you are often faced with a trade-off between benefits and costs (e.g. time, money, inconvenience, effort). Initiatives often aim to reduce various types of emissions (e.g. methane or CO_2), which are greenhouse gases that cause climate change. Below are several suggestions for rules to improve sustainability, but each comes at a cost (e.g. monetary cost, behavioural change, etc.).
On a scale from 1=strongly disagree to 5=strongly agree, please indicate to what extent you would be in favor of such a rule.”

Table S1
Items used for measuring policy support. Standardised Cronbach’s alpha was 0.84.

Items	M/SD
1. Turning more cities into car-free cities relying primarily on public transport, walking, or cycling for transport, which will reduce emissions and pollution. However, it would mean longer travel times for some people.	4.19/1.04
2. Tight regulations on meat and dairy production to reduce methane emissions. However, this means the prices for these products will increase.	4.00/1.16
3. Switching to predominantly vegetarian or vegan options in public canteens & cafeterias, which reduces meat consumption and reduces emissions. However, it means having fewer options to choose from.	4.12/1.19
4. Tight regulations to discourage the use of coal, which will reduce CO_2 emissions. However, this means many people from the coal industry will lose their jobs and have to look for other sources of income.	4.13/1.04
5. Investing in renewable energy technologies to make them cheaper and more effective, which would lead to a reduction of emissions. However, it would mean raising taxes.	4.34/0.93
6. Banning short-haul flights, which will reduce fuel emissions. However, it would lead to longer travel times due to slower alternative transportation options (for example, a train or bus).	3.95/1.20
7. Make flying less attractive by increasing the taxes on flying, which will lead to a reduction of emissions. However, the plane tickets prices will increase.	3.64/1.35
8. Phasing out fossil fuel vehicles to decrease emissions. However, the new vehicles will have higher prices.	4.25/0.95
9. Ban single use plastic packaging, which will reduce plastic pollution. However, the prices of some products will increase.	4.30/0.99
10. Regulating the fashion industry to reduce the environmental impact of cheaply made, mass-produced, imported clothing (known as ”fast fashion“). However, it would lead to increased prices for garments.	4.58/0.78
11. Educating students on climate change and ways to act more sustainably. However, it would mean less time for other subjects.	4.43/0.86

12. Investing more in research on new technology for combating climate change, even if it means decreased investment in other technology.	4.22/0.91

2.5. Description of the environmental initiative

To ensure clarity and credibility, we were careful in framing of what the money “won” for the CO_2 reduction initiative meant. Participants were explicitly informed that we had selected a specific organization with a strong track record in implementing effective greenhouse gas reduction measures, so that their contributions would translate into genuine and direct reductions in CO_2 emissions rather than vague or symbolic actions. At the same time, we deliberately did not disclose the name of the organization to participants. This was done to minimize the risk that prior knowledge, experiences, or attitudes toward a specific organization would influence decisions. Below, we have included the exact description provided to the participants during the instruction phase of the experiment. The experimenter read out these instructions aloud while simultaneously displaying the same text on presentation slides.

Slide 1:

The money you earn for the environmental protection organization today will be donated to an organization that actively promotes climate protection. This means that greenhouse gas emissions are reduced directly and sustainably. To this end, fossil fuels are replaced by renewable energies, and energy-efficient technologies are implemented. The organization runs projects that enable, for example, the expansion of solar energy, hydro and wind power, biogas and biomass, as well as efficient stoves and other measures for direct greenhouse gas reduction.

Slide 2:

Every point you score in today's experiment for the environmental organization will be converted into money and donated to the organization. In this way, you are actively contributing to reducing CO_2 emissions immediately and directly.

2.6. Specifications for the mixed effects models

To analyse the force and response time data with continuous outcomes, we fitted linear mixed-effects models (LMMs) using the lme4 package (Bates et al., 2015). To analyse the choice data with binary outcomes, we fitted logistic mixed-effects models using the package glmmTMB due to its computational efficiency and extended flexibility (Brooks et al., 2017), which allowed our models to converge with a more complex random structure as opposed to lme4. For all models, we included the following variables: recipient (i.e., self or environment), cost level (effort level 1-5 for the effort task, and time level 1-5 for the time task), and reward level (1-5), both modelled as continuous variables. In the same models, we also included all their interactions (i.e., double and triple interactions among the variables), and we added the trial number as a covariate to control for fatigue effects over time.

To aid interpretation of the fixed effects in the linear mixed-effects model, we calculated Cohen’s d for each regressor by dividing the unstandardized regression coefficient (β) by the residual standard deviation of the model (σ). This approach provides a standardized

estimate of effect size for each predictor (Cohen, 1988) and is appropriate for linear models with continuous outcomes (e.g., reaction time). Residual standard deviation was obtained using the sigma() function from the lme4 package (Bates et al., 2015), and effect sizes were computed in R.

2.7. Specifications for the Bayesian analyses for null results

To complement the frequentist analyses, we conducted Bayesian hypothesis tests for all models, yielding null results. These analyses were implemented in R using the brms package (Bürkner, 2017).

We ran Bayesian linear mixed models and multiple regressions that matched the structure of the corresponding frequentist models reported in the main text. Given the lack of established default Bayes factor solutions for linear mixed models, we employed the fractional Bayes factor approach (O'Hagan, 1995). This method uses a small fraction of the information contained in the data to generate minimally informative priors, and then applies the remaining information from the data to compute the Bayes factor. Conceptually, this is simila to using a small part of the data set as a training set to determine reasonable priors, and then using the remaining dataset for hypothesis testing. The advantage of this approach is that it avoids the arbitrariness of specifying priors in advance, while ensuring that Bayes factors are calibrated to the actual data structure and model complexity.

For nonparametric comparisons conducted with Wilcoxon signed-rank tests, we used the JASP software (JASP Team, 2025), which provides a direct Bayesian implementation of these tests. This approach allows the computation of Bayes factors without the need to approximate priors, thereby offering a straightforward and widely accepted method for evaluating evidence in paired-sample designs.

All Bayes factors are reported as BF_{10}, quantifying evidence in favor of the alternative hypothesis relative to the null.

2.8. Computational modeling

2.8.1. Parameterization and priors

Following a hierarchical modeling approach, we assumed that subject and condition specific discounting parameters were distributed as:

$$k_{Self,\,i} = 3 \, * \, \phi^{-1}(\hat{k}_{Self,i})$$
$$\hat{k}_{Self,\,i} \sim N(\mu_{k,\,Self}, \sigma_{k,\,Self})$$

and

$$k_{Env,\,i} = 3 \, * \, \phi^{-1}(\hat{k}_{Self,i} + \delta_{k,i})$$
$$\delta_{k,i} \sim N(\mu_{\delta,\,k}, \sigma_{\delta,\,k}),$$

where i is the subject index, and ϕ^{-1} is the probit transform (the inverse of the cumulative distribution function of the standard normal distribution). This effectively restricted the discounting parameter to lie in the range (0,3). In models with only one discounting parameter, only $k_{Self,i}$ was estimated, and assumed to govern effort discounting in both conditions (*Self* and *Environment*). For the population parameters, we formulated the following priors:

$$\mu_{k,\,Self}, \mu_{\delta,\,k} \sim N(0, 1) \;\; ; \;\; \sigma_{k,\,Self}, \sigma_{\delta,\,k} \sim HalfCauchy(0, 0.2).$$

Similarly, we assumed that subject and condition specific inverse temperature parameters were distributed as:

$$\beta_{Self,\,i} = 50 * \phi^{-1}(\hat{\beta}_{Self,i})$$
$$\hat{\beta}_{Self,\,i} \sim N(\mu_{\beta,\,Self}, \sigma_{\beta,\,Self})$$

and

$$\beta_{Env,\,i} = 50 * \phi^{-1}(\hat{\beta}_{Self,i} + \delta_{\beta,i})$$
$$\delta_{\beta,i} \sim N(\mu_{\delta,\,\beta}, \sigma_{\delta,\,\beta}),$$

where

$$\mu_{\beta,\,Self}, \mu_{\delta,\,\beta} \sim N(0, 1) \;\; ; \;\; \sigma_{\beta,\,Self}, \sigma_{\delta,\,\beta} \sim HalfCauchy(0, 0.2).$$

2.8.2. Hierarchical Bayesian Modeling - posterior predictive checks

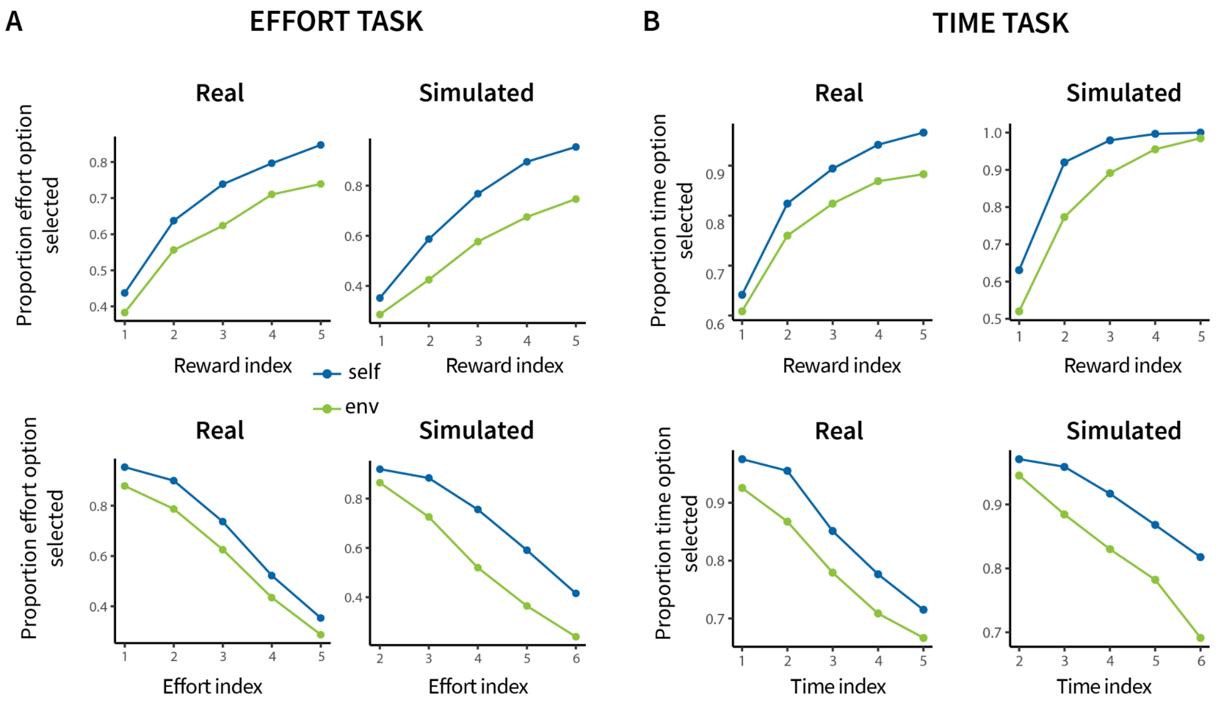

Figure S3. Average proportion of selecting the costly option for the A) effort task and B) time task for the actual data and the simulated data from the winning model (2k2ß parabolic for the effort task and 2k2ß hyperbolic for the time task. Plotted per recipient (self vs environment and reward level in the upper panels and cost level at the lower panels (A. effort, B. time)

2.8.3. Hierarchical Bayesian Modeling - parameter recovery

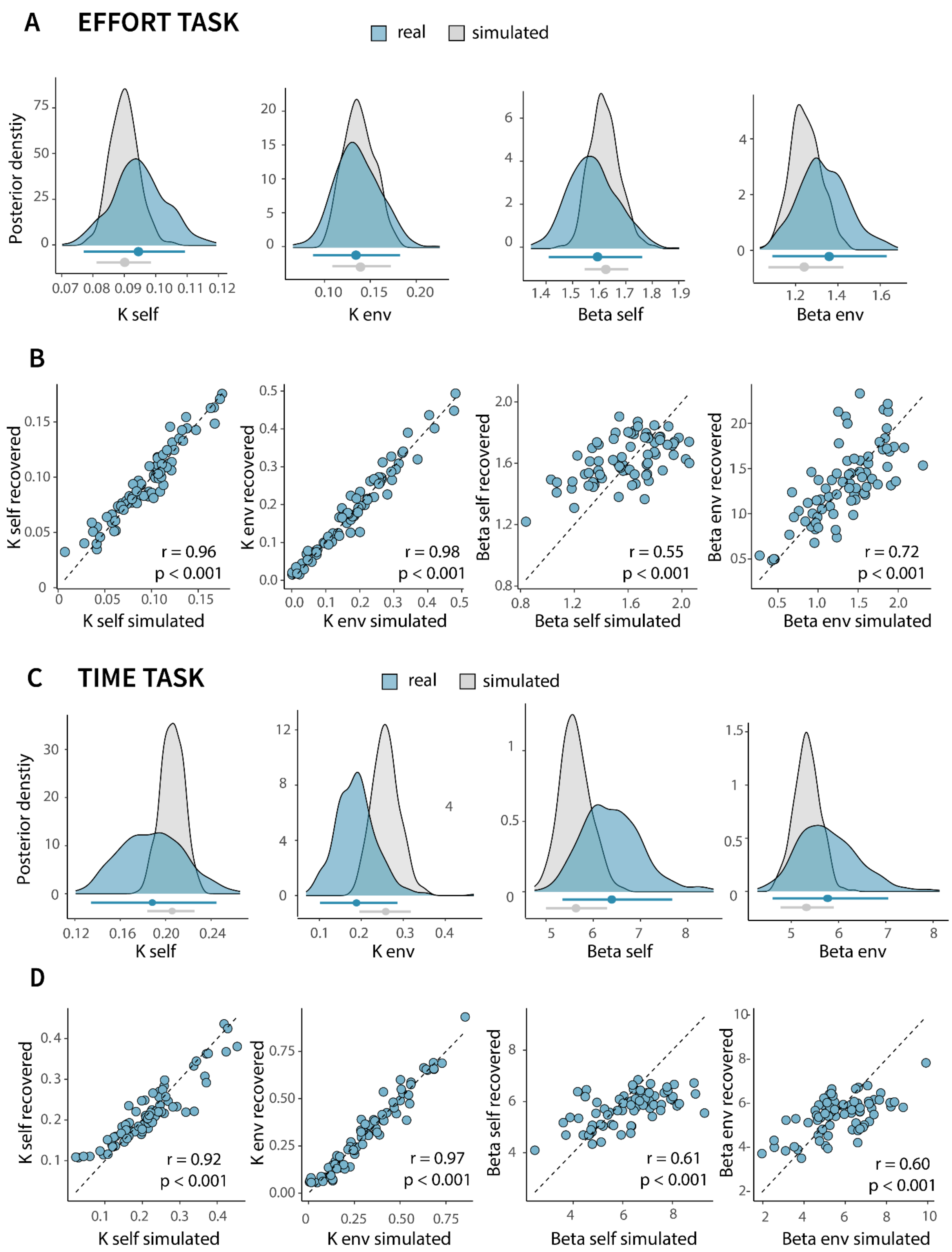

Figure S4. A. & C. Parameter recovery of the group-level posterior distributions for A. the effort task and C. the time task. The blue distribution represents the “ground truth” distribution and the grey distribution the distribution of the simulated dataset. The horizontal lines indicate the group-level 95% HDI of the parameter estimate and the point stands for the

mean of the distribution. B. & D. Pearson’s correlations between the simulated and recovered subject level parameters for the B. effort task and D. time task.

3. Supplementary results

3.1. Distribution of climate change key beliefs and policy support

Regarding the distribution of the climate change key beliefs ratings, it is important to note that we lacked participants with the lowest levels of belief. There were no participants who rated their climate change certainty as lower than 5 on a 1-9 scale, and no participants who reported thinking climate change is not happening or caused mainly by natural causes. The distribution of the policy support measure exhibited a right-skew (mean = 4.18 on a scale from 1 to 5), but the responses were more evenly distributed compared to the majority of the climate change belief subscales.

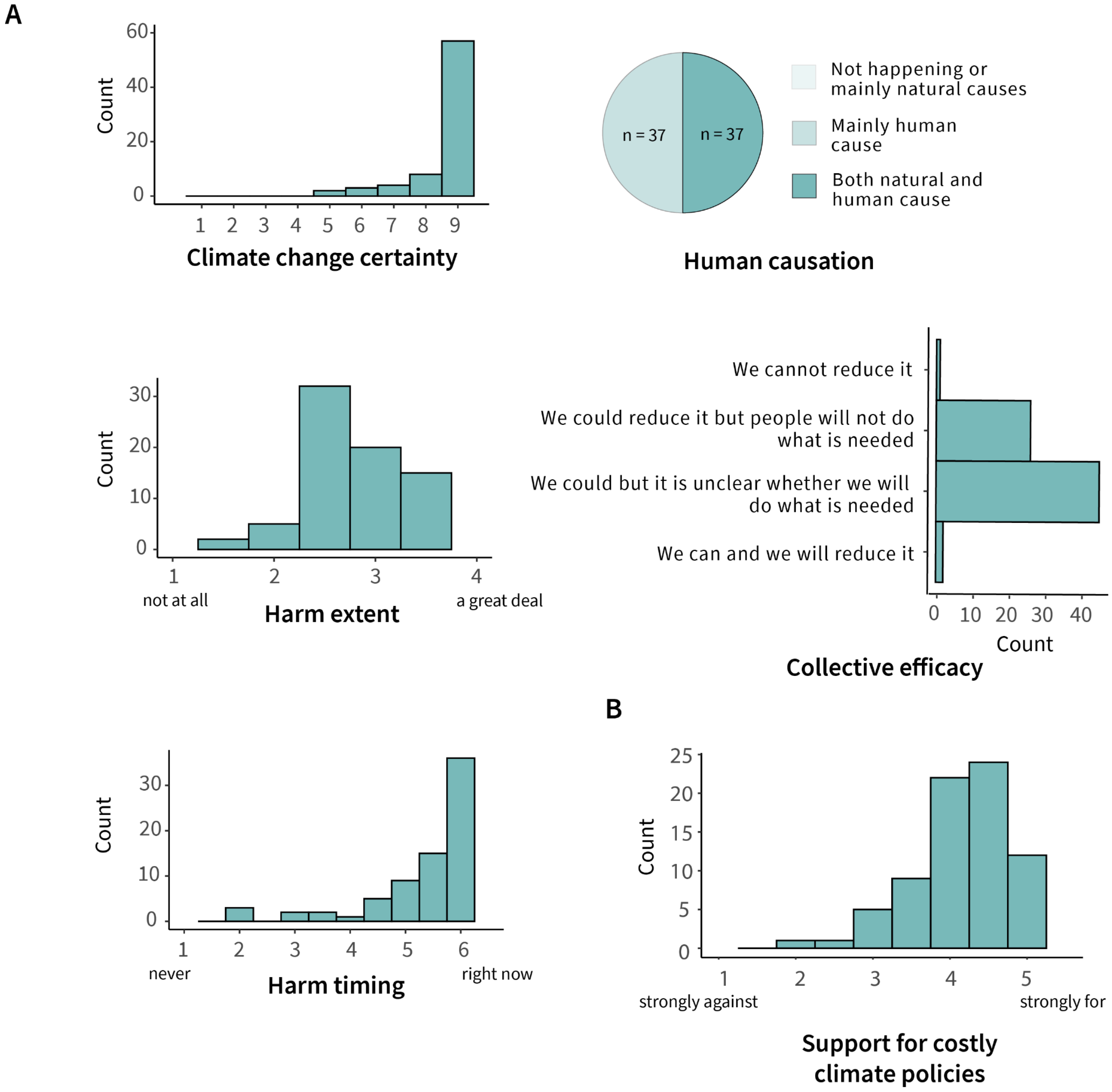

Figure S5. Distribution of the responses to the items used for A. measuring climate change key beliefs: belief certainty (1 item), human causation (1 item), collective efficacy (1 item),

harm timing (average score of 2 items), and harm extent (average score of 7 items). B. policy support.

3.2. Distribution of political orientation and values scores

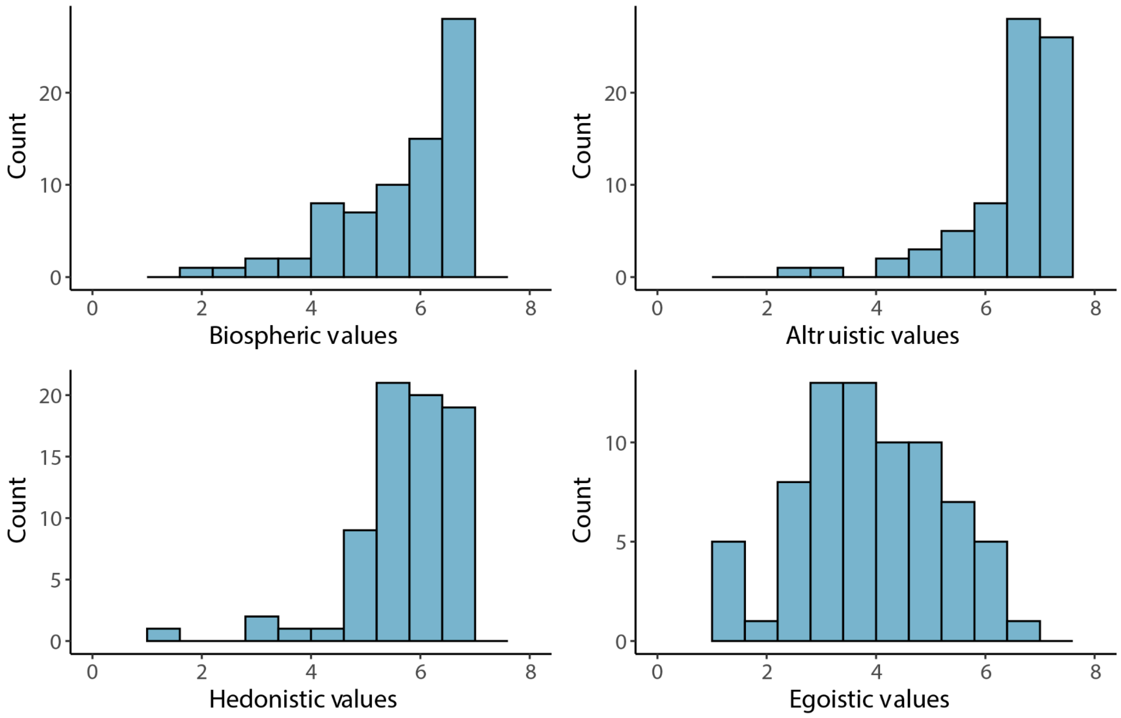

Figure S6. Distribution of the scores for participants’ biospheric, altruistic, hedonistic and egoistic values.

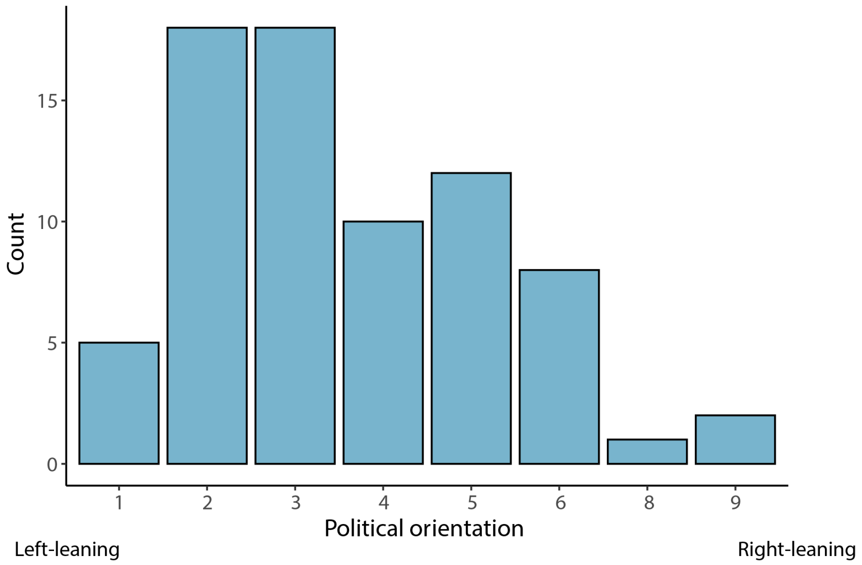

Figure S7. Distribution of participants’ political orientation, measured on a Likert scale from 1 to 9.

3.3. Additional plots for the proportions of costly decisions

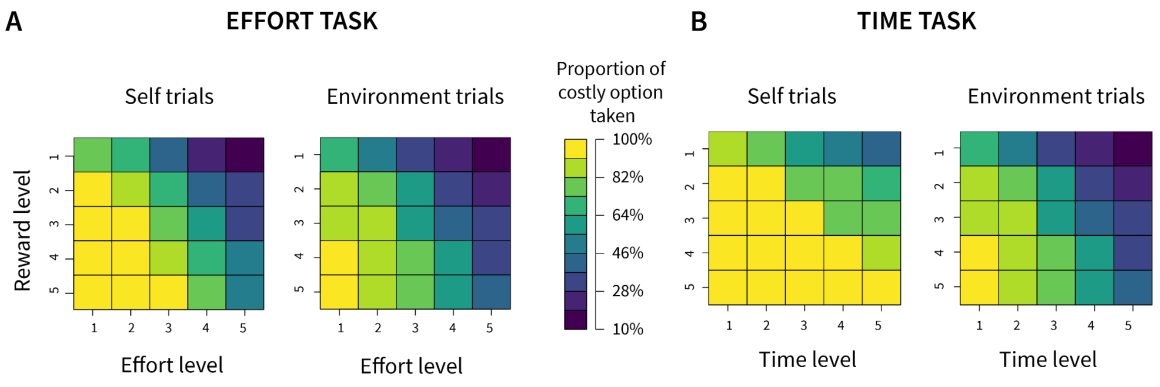

Figure S8. Proportion of costly option taken plotted for each level of effort and reward, separate for self and environmental trials

3.4. Linear mixed models output

Note: we centered reward level, effort level, time level and trial number for all models, following best practices outlined in Brauer & Curtin (2018).

3.4.1. Models with the full sample

Table S2

Effort task: decision<br>glmmTMB(decision ~ recipient * effort * reward + trial + (recipient + effort + reward + recipient:reward | participant code), family = binomial)			
Predictors	*Odds Ratios*	*CI*	*p*
(Intercept)	14.30	7.66 – 26.69	**<0.001**
**recipient [environment]**	**0.32**	**0.15 – 0.70**	**0.004**
**effort level**	**0.15**	**0.12 – 0.18**	**<0.001**
**reward level**	**4.68**	**3.70 – 5.93**	**<0.001**
**trial**	**0.99**	**0.99 – 0.99**	**<0.001**
recipient [environment] × effort level	1.02	0.85 – 1.23	0.805
**recipient [environment] × reward level**	**0.75**	**0.60 – 0.93**	**0.009**
**effort level × reward level**	**0.83**	**0.77 – 0.90**	**<0.001**
recipient [environment] × effort level × reward level	1.04	0.84 – 1.29	0.693
Observations	11071		
Marginal R^2 / Conditional R^2	0.469 / 0.870		

Table S3

Time task: decision

glmmTMB(decision ~ recipient * time * reward + trial + (recipient + time + reward | participant code), data = time_decision, family = binomial)			
Predictors	*Odds Ratios*	*CI*	*p*
(Intercept)	362.03	113.86 – 1151.07	**<0.001**
recipient [environment]	0.44	0.18 – 1.07	0.069
**time level**	**0.30**	**0.23 – 0.39**	**<0.001**
**reward level**	**7.05**	**4.54 – 10.93**	**<0.001**
**trial**	**0.99**	**0.99 – 0.99**	**<0.001**
recipient [environment] × timelevel	1.14	0.93 – 1.39	0.212
**recipient [environment] × reward level**	**0.79**	**0.65 – 0.96**	**0.020**
time level × reward level	0.98	0.89 – 1.08	0.645
recipient [environment] × time level × reward level	1.00	0.89 – 1.12	0.978
Observations	11067		
Marginal R^2 / Conditional R^2	0.285 / 0.904		

Table S4

Effort task: decision duration lmer(decision_time ~ recipient * effort * reward + trial + (recipient + effort + reward + recipient:effort + effort:reward | participant code)			
Predictors	*Estimates*	*CI*	*p*
(Intercept)	1.12	1.06 – 1.17	**<0.001**
recipient [environment]	0.01	-0.01 – 0.03	0.466
**effort level**	**0.06**	**0.05 – 0.07**	**<0.001**
reward level	-0.01	-0.01 – 0.00	0.145
**trial**	**-0.00**	**-0.00 – -0.00**	**<0.001**
recipient [environment] × effort level	-0.01	-0.02 – 0.00	0.152
recipient [environment] × reward level	-0.01	-0.02 – 0.00	0.069
**effort level × reward level**	**0.02**	**0.01 – 0.03**	**<0.001**
recipient [environment] × effort level) × reward level	-0.00	-0.01 – 0.00	0.296
Observations	11071		
Marginal R^2 / Conditional R^2	0.056 / 0.392		

Table S5

Time task: decision duration
lmer(decision_time ~ recipient * time * reward + trial +
(recipient + time + reward + recipient:time | participant code)

Predictors	*Estimates*	*CI*	*p*
(Intercept)	1.04	0.98 – 1.11	**<0.001**
recipient [environment]	0.01	-0.01 – 0.03	0.341
**time level**	**0.04**	**0.03 – 0.05**	**<0.001**
**reward level**	**-0.02**	**-0.03 – -0.01**	**<0.001**
**trial**	**-0.00**	**-0.00 – -0.00**	**<0.001**
recipient [environment] × time level	-0.01	-0.02 – 0.00	0.162
recipient [environment] × reward level	-0.01	-0.01 – 0.00	0.189
**time level × reward level**	**0.01**	**0.00 – 0.01**	**0.012**
recipient [environment] × effort level × reward level	-0.00	-0.01 – 0.01	0.957
Observations	11067		
Marginal R^2 / Conditional R^2	0.022 / 0.405		

Table S6

Effort task: force AUC
lmer(AUC ~ recipient * effort * reward + trial +
(recipient + effort | participant code)

Predictors	*Estimates*	*CI*	*p*
(Intercept)	0.535	0.524 – 0.546	**<0.001**
recipient [environment]	-0.003	-0.007 – 0.001	0.107
**effort level**	**0.070**	**0.067 – 0.073**	**<0.001**
**reward level**	**0.004**	**0.003 – 0.006**	**<0.001**
**trial**	**-0.000**	**-0.000 – -0.000**	**<0.001**
recipient [environment] × effort level	-0.001	-0.003 – 0.001	0.549
recipient [environment] × reward level	-0.001	-0.003 – 0.001	0.194
effort level × reward level	-0.000	-0.001 – 0.001	0.349
recipient [environment] × effort level × reward level	-0.000	-0.001 – 0.001	0.961
Observations	7177		
Marginal R^2 / Conditional R^2	0.626 / 0.789		

Table S7

Effort task: force target reached duration
lmer(target_reached_duration ~ recipient * effort * reward + trial +
(recipient + effort | participant code)

Predictors	*Estimates*	*CI*	*p*
(Intercept)	1.977	1.910 – 2.043	**<0.001**
**recipient [environment]**	**-0.043**	**-0.074 – -0.011**	**0.008**
**effort level**	**-0.149**	**-0.170 – -0.127**	**<0.001**
**reward level**	**0.036**	**0.025 – 0.047**	**<0.001**

**trial**	**-0.001**	**-0.002 – -0.001**	**<0.001**
recipient [environment] × effort level	-0.013	-0.030 – 0.003	0.110
recipient [environment] × reward level	-0.004	-0.020 – 0.012	0.607
effort level × reward level	-0.002	-0.010 – 0.005	0.574
recipient [environment] × effort level × reward level	0.004	-0.007 – 0.015	0.457
**Random Effects**			
Observations	7177		
Marginal R^2 / Conditional R^2	0.148 / 0.433		

3.4.2. Models excluding participants with little to no variability in their behaviour

As per our preregistration, we ran these analyses by excluding participants who show little to no variability in their choices.

Table S8

Effort task: decision
glmmTMB(decision ~ recipient * effort * reward + trial + (recipient + effort + reward + recipient:reward | participant code), family = binomial)

Predictors	*Odds Ratios*	*CI*	*p*
(Intercept)	9.76	5.67 – 16.79	**<0.001**
**recipient [environment]**	**0.31**	**0.14 – 0.69**	**0.004**
**effort level**	**0.14**	**0.12 – 0.18**	**<0.001**
**reward level**	**4.44**	**3.54 – 5.58**	**<0.001**
**trial**	**0.99**	**0.99 – 0.99**	**<0.001**
recipient [environment] × effort level	1.02	0.85 – 1.23	0.839
**recipient [environment] × reward level**	**0.78**	**0.63 – 0.96**	**0.018**
**effort level × reward level**	**0.83**	**0.76 – 0.89**	**<0.001**
recipient [environment] × effort level × reward level	1.02	0.92 – 1.14	0.702
Observations	10321		
Marginal R^2 / Conditional R^2	0.512 / 0.859		

Table S9

Time task: decision
glmmTMB(decision ~ recipient * time * reward + trial + (recipient + time + reward | participant code), data = time_decision, family = binomial)

Predictors	*Odds Ratios*	*CI*	*p*
(Intercept)	362.03	113.86 – 1151.07	**<0.001**
**recipient [environment]**	**0.29**	**0.12 – 0.72**	**0.008**
**time level**	**0.24**	**0.19 – 0.30**	**<0.001**

Predictors	Odds Ratios	CI	p
**reward level**	**6.00**	**4.09 – 8.81**	**<0.001**
**trial**	**0.99**	**0.99 – 0.99**	**<0.001**
recipient [environment] × timelevel	1.13	0.93 – 1.39	0.222
**recipient [environment] × reward level**	**0.79**	**0.65 – 0.96**	**0.019**
time level × reward level	0.98	0.89 – 1.08	0.649
recipient [environment] × time level × reward level	0.99	0.88 – 1.12	0.903
Observations	6574		
Marginal R^2 / Conditional R^2	0.439 / 0.857		

3.4.3. Models excluding people with doubts about being observed

Table S10

Effort task: decision<br>glmmTMB(decision ~ recipient * effort * reward + trial + (recipient + effort + reward + recipient:reward | participant code), family = binomial)			
Predictors	*Odds Ratios*	*CI*	*p*
(Intercept)	13.65	6.65 – 28.02	**<0.001**
**recipient [environment]**	**0.33**	**0.14 – 0.80**	**0.015**
**effort level**	**0.15**	**0.12 – 0.18**	**<0.001**
**reward level**	**4.86**	**3.71 – 6.36**	**<0.001**
**trial**	**0.99**	**0.99 – 0.99**	**<0.001**
recipient [environment] × effort level	0.92	0.76 – 1.11	0.368
**recipient [environment] × reward level**	**0.80**	**0.64 – 0.99**	**0.042**
**effort level × reward level**	**0.84**	**0.78 – 0.91**	**<0.001**
recipient [environment] × effort level × reward level	0.98	0.88 – 1.10	0.730
Observations	9429		
Marginal R^2 / Conditional R^2	0.452 / 0.883		

Table S11

Time task: decision<br>glmmTMB(decision ~ recipient * time * reward + trial + (recipient + time + reward | participant code), data = time_decision, family = binomial)			
Predictors	*Odds Ratios*	*CI*	*p*
(Intercept)	270.94	81.95 – 895.79	**<0.001**
**recipient [environment]**	**0.36**	**0.14 – 0.91**	**0.031**
**time level**	**0.28**	**0.22 – 0.37**	**<0.001**
**reward level**	**6.94**	**4.41 – 10.93**	**<0.001**
**trial**	**0.99**	**0.99 – 0.99**	**<0.001**
recipient [environment] × timelevel	1.11	0.91 – 1.37	0.309
**recipient [environment] × reward level**	**0.79**	**0.65 – 0.97**	**0.026**
time level × reward level	1.01	0.91 – 1.11	0.915

recipient [environment] × time level × reward level	0.97	0.86 – 1.10	0.624
Observations	9424		
Marginal R^2 / Conditional R^2	0.295 / 0.905		

3.5. Climate change key beliefs and discounting

Table S12

Effort task<br>rlm(effort_k_diff ~ cc_harm_timing + cc_harm_extent + cc_certainty + cc_cause + cc_collective_efficacy)			
Predictors	***Estimates***	***CI***	***p***
(Intercept)	-0.23	-0.37 – -0.09	**0.002**
cc harm timing	0.01	-0.01 – 0.03	0.196
cc harm extent	0.01	-0.03 – 0.05	0.505
cc belief certainty	0.02	-0.00 – 0.03	0.052
cc cause	-0.01	-0.04 – 0.02	0.516
cc collective efficacy	-0.00	-0.03 – 0.02	0.917
Observations	74		

Table S13

Time task<br>rlm(time_k_diff ~ cc_harm_timing + cc_harm_extent + cc_certainty + cc_cause + cc_collective_efficacy)			
Predictors	***Estimates***	***CI***	***p***
(Intercept)	-0.21	-0.50 – 0.07	0.136
cc harm timing	0.01	-0.02 – 0.05	0.499
cc harm extent	-0.06	-0.14 – 0.03	0.177
cc belief certainty	0.03	-0.01 – 0.06	0.095
cc cause	0.01	-0.05 – 0.07	0.758
cc collective efficacy	0.01	-0.04 – 0.07	0.628
Observations	74		

3.6. Ratings of effort and time levels pre- and post-testing session

Participants experienced each effort level and were be asked to complete two items from the NASA Task Load Index (Hart & Staveland, 1988); Physical Demand: “How physically demanding was the task?”, Effort: “How hard did you have to work to accomplish your level of performance?”) and one additional question (Unpleasantness: “How unpleasant was it for you?”) for each of the 5 effort levels.

Table S14

Items used for rating the effort levels pre- and post the effort task. Measured on a scale from 1 to 100.

Effort level	Testing point	Physical demand	Unpleasantness	Effort
40%	pre	29.23	24.91	52.58
	post	33.18	35.41	52.79
50%	pre	38.96	33.00	56.41
	post	45.75	46.82	59.68
60%	pre	47.68	44.61	65.59
	post	57.85	58.45	69.56
70%	pre	58.80	55.30	72.51
	post	72.29	72.08	81.23
80%	pre	72.95	67.82	82.01
	post	85.78	84.22	92.12

Participants also experienced each time level and were asked to complete three items: “How long did the passing of time seem to you?”, “How frustrating was it to wait for the time to pass?”, ”'How boring was it to wait for the time to pass?”

Table S15
Items used for rating the time levels pre- and post the time task. Measured on a scale from 1 to 100.

Time level	Testing point	Time demand	Frustrating	Boring
1	pre	25.38	13.15	19.20
	post	24.41	19.41	20.93
2	pre	32.54	21.80	26.65
	post	34.04	26.82	31.79
3	pre	38.97	28.74	32.82
	post	48.03	36.93	44.58
4	pre	49.03	33.85	42.08
	post	58.52	45.03	52.38
5	pre	58.89	40.64	50.89
	post	67.22	50.51	61.27

3.7. Preregistered analyses not included due to being suboptimal

3.7.1. LMMs without trial number as covariate and with simpler error term

Table S16

**Effort task: decision**<br>**glmmTMB(decision ~ recipient * effort * reward + (1 | participant code), family = binomial)**			
Predictors	*Odds Ratios*	*CI*	*p*
(Intercept)	6.87	4.64 – 10.16	**<0.001**
**recipient [environment]**	**0.34**	**0.29 – 0.39**	**<0.001**
**effort level**	**0.23**	**0.21 – 0.25**	**<0.001**
**reward level**	**2.73**	**2.52 – 2.96**	**<0.001**
**recipient [environment] × effort level**	**1.48**	**1.34 – 1.64**	**<0.001**
**recipient [environment] × reward level**	**0.69**	**0.63 – 0.76**	**<0.001**
**effort level × reward level**	**0.86**	**0.81 – 0.90**	**<0.001**
**recipient [environment] × effort level × reward level**	**1.18**	**1.10 – 1.26**	**<0.001**
Observations	11071		
Marginal R^2 / Conditional R^2	0.460 / 0.701		

Table S17

**Effort task: decision duration**<br>**lmer(decision_time ~ recipient * effort * reward + (1 | participant code))**			
Predictors	*Odds Ratios*	*CI*	*p*
(Intercept)	1.12	1.06 – 1.17	**<0.001**
recipient [environment]	0.01	-0.00 – 0.02	0.070
**effort level**	**0.06**	**0.05 – 0.07**	**<0.001**
reward level	-0.01	-0.01 – 0.00	0.104
**recipient [environment] × effort level**	**-0.01**	**-0.02 – -0.00**	**0.046**
**recipient [environment] × reward level**	**-0.01**	**-0.02 – -0.00**	**0.044**
**effort level × reward level**	**0.02**	**0.02 – 0.02**	**<0.001**
recipient [environment] × effort level × reward level	-0.01	-0.01 – 0.00	0.119
Observations	11071		
Marginal R^2 / Conditional R^2	0.042 / 0.333		

Table S18

**Effort task: force AUC**<br>**lmer(AUC ~ recipient * effort * reward + (1 | participant code))**			
Predictors	*Estimates*	*CI*	*p*

(Intercept)	0.524	0.514 – 0.535	**<0.001**
recipient [environment]	-0.002	-0.005 – 0.001	0.157
**effort level**	**0.070**	**0.069 – 0.071**	**<0.001**
**reward level**	**0.004**	**0.002 – 0.005**	**<0.001**
recipient [environment] × effort level	-0.000	-0.002 – 0.002	0.706
recipient [environment] × reward level	-0.001	-0.004 – 0.001	0.183
effort level × reward level	-0.001	-0.002 – 0.000	0.142
recipient [environment] × effort level × reward level	-0.000	-0.002 – 0.001	0.853
Observations	7177		
Marginal R^2 / Conditional R^2	0.621 / 0.770		

Table S19

Time task: decision
glmmTMB(decision ~ recipient * time * reward + (1 | participant code), family = binomial)

Predictors	*Odds Ratios*	*CI*	*p*
(Intercept)	92.76	44.69 – 192.57	**<0.001**
**recipient [environment]**	**0.23**	**0.19 – 0.29**	**<0.001**
**time level**	**0.30**	**0.26 – 0.34**	**<0.001**
**reward level**	**3.74**	**3.29 – 4.26**	**<0.001**
**recipient [environment] × time level**	**1.67**	**1.45 – 1.92**	**<0.001**
**recipient [environment] × reward level**	**0.53**	**0.46 – 0.61**	**<0.001**
**time level × reward level**	**0.90**	**0.83 – 0.97**	**0.010**
**recipient [environment] × time level × reward level**	**1.18**	**1.08 – 1.30**	**<0.001**
Observations	11071		
Marginal R^2 / Conditional R^2	0.290 / 0.799		

Table S20

Time task: decision duration
lmer(decision_time ~ recipient * time * reward + (1 | participant code)

Predictors	*Estimates*	*CI*	*p*
(Intercept)	1.04	0.98 – 1.10	**<0.001**
recipient [environment]	0.01	-0.00 – 0.02	0.134
**time level**	**0.04**	**0.03 – 0.05**	**<0.001**
**reward level**	**-0.02**	**-0.03 – -0.01**	**<0.001**
recipient [environment] × time level	-0.01	-0.02 – 0.00	0.124
recipient [environment] × reward level	-0.01	-0.02 – 0.00	0.158
**time level × reward level**	**0.01**	**0.00 – 0.01**	**0.013**
recipient [environment] × effort level × reward level	-0.00	-0.01 – 0.01	0.771

Observations	11067
Marginal R^2 / Conditional R^2	0.022 / 0.381

3.7.2. Low-cost hypothesis

Here, as per our preregistration, we selected only trials where participants were making choices for the environment to see whether there is an interaction between climate change and the cost.

Table S21

**Effort task: decision<br>glmmTMB(decision ~ effort_level * reward_level + trial + effort_level:cc_beliefs + (effort_level + reward_level | participant_code), family = binomial)**			
Predictors	Odds Ratios	CI	p
(Intercept)	4.72	2.13 – 10.46	<0.001
**effort level**	**0.14**	**0.11 – 0.17**	**<0.001**
**reward level**	**3.73**	**2.90 – 4.81**	**<0.001**
**trial**	**0.99**	**0.99 – 0.99**	**<0.001**
**effort level × reward level**	**0.83**	**0.77 – 0.90**	**<0.001**
effort level × climate change beliefs	0.90	0.67 – 1.21	0.494
Observations	5512		
Marginal R^2 / Conditional R^2	0.407 / 0.886		

Table S22

**Time task: decision<br>glmmTMB(decision ~ time_level * reward_level + trial + time_level:cc_beliefs + (effort_level + reward_level | participant_code), family = binomial)**			
Predictors	Odds Ratios	CI	p
(Intercept)	178.84	42.91 – 745.36	**<0.001**
**time level**	**0.31**	**0.24 – 0.41**	**<0.001**
**reward level**	**6.82**	**4.27 – 10.89**	**<0.001**
**trial**	**0.99**	**0.98 – 0.99**	**<0.001**

**time level× reward level**	**0.94**	**0.86 – 1.02**	**0.148**
time **level**× climate change belief	0.98	0.78 – 1.25	0.898
Observations	5509		
Marginal R^2 / Conditional R^2	0.251 / 0.921		

3.7.3. Linear regression between policy support and discounting difference

Following our preregistration, we conducted a simple linear regression to examine whether policy support predicted the difference in discounting for each task. Due to the presence of outliers in the discounting difference for both time and effort, winsorizing was used.

For the effort task, the model was significant, $F(1, 72) = 14.49$, $p < 0.001$, and explained approximately 16.8% of the variance in effort discounting differene, $R^2 = 0.17$, adjusted $R^2 = 0.16$. Policy support was a significant positive predictor of the difference in effort discounting, $b = 0.17$, $t(72) = 3.81$, $p < 0.001$, with a 95% confidence interval of [0.08, 0.26].

For the time task, the model was significant, $F(1, 72) = 6.22$, $p = 0.015$, and explained approximately 8% of the variance in time discounting differene, $R^2 = 0.08$, adjusted $R^2 = 0.07$. Policy support was a significant positive predictor of the difference in temporal discounting, $b = 0.18$, $t(72) = 2.49$, $p = 0.015$, with a 95% confidence interval of [0.04-0.32].

3.8. Exploratory modeling analyses

We also conducted exploratory modelling analysis inspired by conference conversations about our study and the analysis approach. The most important added aspect was that we tested models incorporating reward sensitivity parameters with either a linear or a logarithmic function

Effort task:

- Linear reward and parabolic effort discounting:

$$SV = \rho * R - k * E^{2}$$

- Logarithmic reward and parabolic effort discounting:

$$SV = \rho * log(R) - k * E^{2}$$

Time task:

- Linear reward and hyperbolic time discounting:

$$SV = \rho * R * \frac{1}{1 + k * T(t)}$$

- Logarithmic reward and hyperbolic discounting:

$$SV = \rho * log(R) * \frac{1}{1 + k * T}$$

Table S23
LOOIC values for the models incorporating reward sensitivity, effort discounting and a choice stochasticity parameters.

**EFFORT**	Linear ρ	Log ρ	**TIME**	Linear ρ	Log ρ
	Parabolic k	Parabolic k		Hyper k	Hyper k
**Model**	**LOOIC**	**LOOIC**		**LOOIC**	**LOOIC**
1r1k1ß	7866.6	7627.8		4964.1	4946.6
1r2k1ß	6634.1	6381.7		3989.4	3988.2
1r1k2ß	7306.6	7034.6		4423.3	4425.8
1r2k2ß	6523.1	**6317.1**		3993.3	**3986.9**
2r1k1ß	6482.9	6583.1		4036.9	4007.6
2r2k1ß	6467.5	6395.9		4048.4	4013.8
2r1k2ß	6461.2	6378.6		4026.2	4003.9
2r2k2ß	6455.4	6382.7		4033.8	4012.9

The winning model for the effort task was modeling reward using a logarithmic function with the same reward sensitivity parameter for self and environmental outcomes, effort discounting with a parabolic function using separate parameters for self and environmental outcomes, and separate parameters for choice stochasticity. However, our model validation procedure showed very poor parameter retrieval (i.e., there was low correlation between known “ground truth” parameters and estimated parameters, indicating high estimation error). The most likely explanation of this issue is non-identifiability that arises when the reward-sensitivity parameter ρ is introduced. This can be seen when one considers the softmax function, which transforms subjective values to choice probabilities:

$$P\ (costly\ option)\ =\ \frac{1}{1+\ exp(-\ ß\ (SV\ costly\ option\ -\ SV\ no\ cost\ option)}$$

For the model with logarithmic reward and parabolic effort discounting, the term within the exponential function can be expanded to

$$-\ \beta(\rho\ *\ log(R)\ -\ k\ *\ E^{2}).$$

One can now choose an arbitrary constant c, and define $\widehat{\beta} = c\ *\ \beta$, $\widehat{\rho} = \frac{\rho}{c}$, and $\widehat{k} = \frac{k}{c}$. Then one gets:

$$- \hat{\beta}(\hat{\rho} * log(R) - \hat{k} * E^2) = - c\beta(\frac{\rho}{c} * log(R) - \frac{k}{c} * E^2) = - \beta(\rho * log(R) - k * E^2)$$

Thus, the parameter configuration $\hat{\beta}$, $\hat{\rho}$, and $\hat{k}$ produces the same likelihood as β, ρ, and k, resulting in non-identifiability.

We then explored whether removing the choice stochasticity parameter and investigating models with only reward sensitivity (ρ) and cost discounting (k) parameters would improve model fit (compared to the original pre-registered models) and would show good identifiability (in contrast to the models above). For that, we used a logarithmic reward sensitivity and parabolic effort discounting for the effort task and hyperbolic time discounting for the time task. These models showed good identifiability and provided better fit than the original models for the effort task, but worse for the time task. We therefore decided to keep the original preregistered models for the main manuscript analysis.

Table S24
LOOIC values for the models incorporating reward sensitivity and effort discounting but without a choice stochasticity

**Model**	**EFFORT**	**TIME**
	LOOIC	LOOIC
1r1k	7819.2	6698.6
1r2k	6582.4	6283.2
2r1k	6463.3	6167.5
2r2k	**6425.3**	**6160.7**

The models for the effort task provided better fit than the original preregistered models, while the models for the time task were worse. Smaller values indicate better fit.

References

Bates, D., Mächler, M., Bolker, B., & Walker, S. (2015). Fitting Linear Mixed-Effects Models Using lme4. *Journal of Statistical Software*, *67*(1), 1–48. https://doi.org/10.18637/jss.v067.i01

Bock, O., Baetge, I., & Nicklisch, A. (2014). hroot: Hamburg Registration and Organization Online Tool. *European Economic Review*, *71*, 117–120. https://doi.org/10.1016/j.euroecorev.2014.07.003

Brooks, M. E., Kristensen, K., Benthem, K. J. van, Magnusson, A., Berg, C. W., Nielsen, A.,

Skaug, H. J., Maechler, M., & Bolker, B. M. (2017). glmmTMB Balances Speed and Flexibility Among Packages for Zero-inflated Generalized Linear Mixed Modeling. *The R Journal*, *9*(2), 378–400. https://doi.org/10.32614/RJ-2017-066

Bürkner, P.-C. (2017). brms: An R Package for Bayesian Multilevel Models Using Stan. *Journal of Statistical Software*, *80*, 1–28. https://doi.org/10.18637/jss.v080.i01

Cohen, J. (1988). *Statistical Power Analysis for the Behavioral Sciences* (2nd ed.). Routledge. https://doi.org/10.4324/9780203771587

Hart, S. G., & Staveland, L. E. (1988). Development of NASA-TLX (Task Load Index): Results of Empirical and Theoretical Research. In P. A. Hancock & N. Meshkati (Eds.), *Advances in Psychology* (Vol. 52, pp. 139–183). North-Holland. https://doi.org/10.1016/S0166-4115(08)62386-9

JASP Team. (2025). *JASP (Version 0.95.1)[Computer software]*. https://jasp-stats.org/

O'Hagan, A. (1995). Fractional Bayes Factors for Model Comparison. *Journal of the Royal Statistical Society: Series B (Methodological)*, *57*(1), 99–118. https://doi.org/10.1111/j.2517-6161.1995.tb02017.x
